# Cobalt(III) as a Stable and Inert Mediator Ion between NTA and His6-Tagged Proteins**

**DOI:** 10.1002/anie.201210317

**Published:** 2013-06-05

**Authors:** Seraphine V Wegner, Joachim P Spatz

**Affiliations:** Abt. Neue Materialieln und Biosysteme, Max-Planck-Institut für Intelligente SystemeHeisenbergstrasse 3, 70596, Stuttgart (Germany) and Abt. Biophysikalische Chemie, Universität Heidelberg(Germany)

The Ni^2+^-mediated interaction between the hexahistidine tag (His6-tag) and nitrilotriacetic acid (NTA) has been shown to be a flexible and reliable way to selectively bind recombinant proteins to NTA-functionalized molecules and materials. The small size of the tag, the site-specific interaction, and the very mild conditions for this interaction, which do not interfere with the native activity of the proteins make the system widely applicable and are the reason for the large library of existing His-tagged proteins. Though initially developed for the purification of recombinant proteins,^1^–^4^ this method has been extended to numerous other applications such as the specific immobilization of proteins on protein chips,^5^, ^6^ the incorporation of active proteins in nanomaterials^7^, ^8^ and on surfaces,^9^, ^10^ the labeling of proteins with fluorophores,^11^–^14^ and the specific conjugation of biomolecules with proteins.^15^–^17^

In the [Ni^II^NTA(His6-tag)] complex, the Ni^2+^ ion is in an octahedral coordination environment, where four coordination sites are occupied by NTA and two by the His6-tag. Similarly, other divalent ions such as Co^2+^, Cu^2+^, and Zn^2+^ can also be used as mediator ions between the His6-tag and NTA with similar affinity.^2^ One drawback of this technology is that the affinity between the NTA and the His6-tag protein mediated by Ni^2+^ or the other ions listed above is usually only in the micromolar range, which is not tight enough for certain applications. Another limitation is that these complexes are kinetically labile and thus undergo rapid ligand exchange. Consequently, chelators such as imidazole and EDTA (ethylenediaminetetraacetic acid) quickly disturb the complex. While this reversible character is favorable for applications such as protein purification, it is problematic if a permanent interaction is desired. For the immobilization of proteins to surfaces, longer His-tags such as the His10-tag are preferred due to better binding. To date, researchers have also proposed the addition of multiple NTA groups to increase affinity^18^–^20^ or forming covalent bonds through photo-chemical^21^, ^22^ (or other less specific)^23^, ^24^ reactions to achieve a permanent interaction, but these approaches typically require complex syntheses and are often unspecific.

To overcome these limitations, a specific, more stable, and kinetically inert interaction between the His6-tag and the NTA group is desirable. Therefore, we propose to use Co^3+^ as the mediator ion for this interaction, which, as a d^6^ ion, forms low-spin octahedral paramagnetic complexes (e_g_^6^t_2g_^0^) that have two advantages: 1) Co^3+^ complexes have significantly higher formation constants than Co^2+^ and Ni^2+^ complexes with similar coordination environments. In particular, Co^3+^ complexes with standard aminopolycarboxylic acids have formation constants that are about 20 orders of magnitudes higher than the Co^2+^ analogues;^25^, ^26^ and 2) Co^3+^ complexes are exchange-inert as they undergo only very slow ligand exchange in their primary coordination sphere. For instance, the aqua complex of Co^2+^ has an exchange rate of 3×10^6^ s^−1^ while the Co^3+^ complex has an exchange rate of less than 10^−6^ s^−1^ (Scheme 1).^27^ The idea to use Co^3+^ as a mediator ion between NTA and the His6-tag has been inspired by studies on metalloenzymes where the substitution of the native metal ion by exchange-inert metal ions such as Co^3+^ and Cr^3+^ has been particularly useful in elucidating enzymatic mechanisms, differentiating between multiple metal sites in an enzyme, and providing insight into the local environment of the metal binding pocket and overall structure.^28^, ^29^ In addition, complexes of bioactive ligands with exchange-inert metal ions have been used as prodrugs that are only activated after entering the cell.^30^, ^31^ Because the formation of Co^3+^ complexes is very slow, an indirect preparation is preferred in which the complex with the exchange-labile divalent ion is formed and then the metal center is oxidized in situ (Scheme 1).

**Scheme 1:**
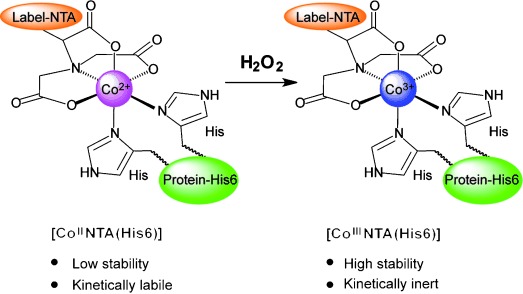
Using Co^3+^ as the mediator ion between NTA and His6-tagged proteins instead of the commonly used Co^2+^ and Ni^2+^ ions leads to the formation a complex that is drastically more stable in such a coordination environment and is inert to ligand-exchange reactions. The Co^3+^ complex can be obtained through the oxidation of the Co^2+^ complex by H_2_O_2_.

Analogously, we also adopted this indirect preparation method to obtain the Co^3+^ complex with NTA and His6-protein, [Co^III^NTA(His6-GFP)]. To test this approach, His6-GFP (N-terminally His6-tagged green fluorescent protein) was immobilized on Co^2+^-loaded NTA agarose beads and aliquots of these beads were incubated with various amounts of H_2_O_2_ for 1 hour. Subsequently 250 mm imidazole was added to each sample and the GFP fluorescence in the supernatant was measured (Figure 1). Similar to His6-tagged proteins on Ni^2+^-NTA beads, the His6-GFP bound to the Co^2+^ center is eluted quickly in the presence of 250 mm imidazole,^2^ while the protein bound to the exchange-inert Co^3+^ center is not. Less and less protein is eluted with increasing H_2_O_2_ concentrations and above 10 mm H_2_O_2_ almost no protein is eluted. The oxidation of Co^2+^ to Co^3+^ on the beads with H_2_O_2_ can also be observed by the color change of the beads from pink to purple (Figure S1 in the Supporting Information). Similarly, when the model complex, [Co^II^NTA(His6-peptide)] is oxidized with H_2_O_2_ in solution, a characteristic d–d transition peak for Co^3+^ is observed in the UV/Vis spectrum at 542 nm (Figure S2 in the Supporting Information). This peak is blue-shifted compared to the analogous peak for [Co^III^NTA] (*λ*_max_=554 nm), as expected, due to the increased number of nitrogen donor ligands. A similar shift is observed for [Co^III^NTA(imidazole)_2_] (*λ*_max_=543 nm). The formation of the [Co^III^NTA(His6-peptide)] complex was further confirmed by ESI-MS. Four other His6-tagged proteins were also successfully immobilized on the Co^3+^-NTA agarose beads using 20 mm H_2_O_2_, which shows the generality of this approach (Figure S3 in the Supporting Information).

**Figure 1:**
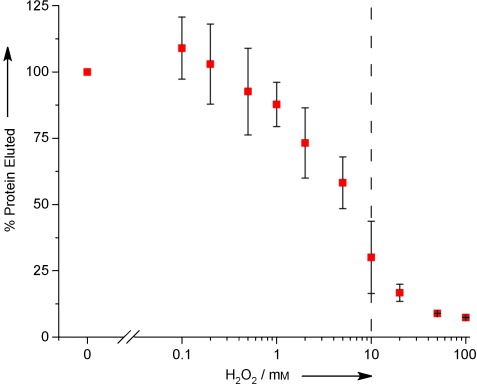
Co^2+^-NTA agarose beads with immobilized His6-GFP were incubated with solutions having various concentrations of hydrogen peroxide for 1 hour. Then the beads were treated with 250 mm imidazole and the eluted His6-GFP was quantified. His6-GFP bound to a Co^2+^ center is eluted while His6-GFP bound to a Co^3+^ center is not.

Secondly, the kinetic inertness of the Co^3+^-mediated interaction between NTA and His6-tag was evaluated. To determine this, the amount of His6-GFP that elutes from the beads described above by means of the ligand-exchange reaction with imidazole was monitored over 16 days (Figure S4 in the Supporting Information). The initial dissociation rate was approximated to first-order reaction kinetics and the observed rate constant for dissociation is calculated to be (1.12±0.14)×10^−6^ s^−1^ at 25 °C. For comparison, the dissociation rate constant for the [Ni^II^NTA (His6-peptide)] complex has been reported to be 1.8 s^−1^.^20^ Thus the [Co^III^NTA(His6-peptide)] complex has a half-life of 7.1 days at room temperature in the presence of 250 mm imidazole, conditions under which the homologous Co^2+^ and Ni^2+^ complexes are transient.

The Co^3+^-mediated interaction between NTA and His6-tag is also very inert towards ligand exchange with strong chelators and reactions with reducing agents. To demonstrate this, aliquots of agarose beads with immobilized [Co^II^NTA(His6-GFP)] and [Co^III^NTA(His6-GFP)] were incubated with either the strong chelators EDTA and NTA (25 mm) or with reducing agents commonly used in protein chemistry—DTT (dithiothreitol), TCEP (tris(2-carboxyethyl)phosphine), cysteamine, and ascorbate (1 mm)—in combination with 250 mm imidazole. After 1 and 24 h of incubation the amount of His6-GFP eluted from the beads was measured (Figure 2 and Figure S5 in the Supporting Information). When His6-GFP is bound to Co^3+^ centers, no significant increase in the eluted His6-GFP is observed upon incubation with the tested chelators or reducing agents. In contrast, similar to what has been reported in the literature for the Ni^2+^-NTA interaction with His6-tagged proteins,^2^ all His6-GFP bound to Co^2+^ beads is eluted under the same conditions and the strong chelators remove the Co^2+^ from the beads as observed by the color change of the beads from pink to white. The Co^3+^-mediated interaction between His6-GFP and the NTA beads was also not disturbed under acidic conditions (pH 3.5) but could be mostly reversed by treatment with 100 mm of ascorbate for 1 day by the reduction to Co^2+^ (Figure S6 in the Supporting Information). Moreover, the agarose beads with His6-GFP bound to Co^3+^ centers were also stable in cell culture for 24 h. (Figure S7 in the Supporting Information). This shows that the [Co^III^NTA(His6-GFP)] complex is very inert towards disruption by strong chelators and reduction back to Co^2+^.

**Figure 2:**
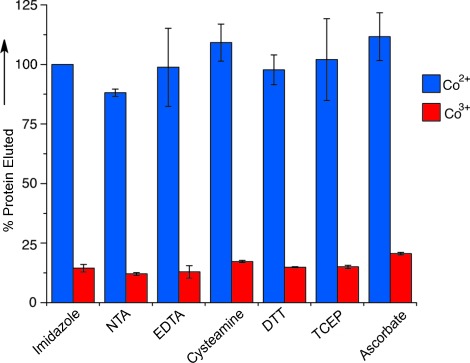
Chemical reactivity of [Co^III^NTA(His6-GFP)]. NTA beads with immobilized His6-GFP at Co^2+^ and Co^3+^ centers were incubated with imidazole (250 mm), the chelators NTA and EDTA (25 mm), and the reducing agents cysteamine, DTT, TCEP, and ascorbate (1 mm) in combination with 250 mm imidazole for 1 hour and the amount of eluted His6-GFP was measured. Due to the kinetic inertness of the Co^3+^ centers, His6-GFP is not eluted when bound to the beads as [Co^III^NTA(His6-GFP)].

The [Co^III^NTA(His6-GFP)] complexes are so inert towards ligand exchange that they can even be passed through a Ni-NTA column without disturbing the complexes; we observed that these complexes have a lower affinity towards this column than His6-tagged protein alone. This can be explained by the fact that some of the histidines in the His6-tag are permanently coordinated to the Co^3+^ center and are no longer available to interact with the Ni-NTA in the column. This stability solves a common problem in protein labeling. As labeling efficiencies are usually not 100 %, it is critical to be able to separate reacted and unreacted species. While it is easy to separate the unreacted small molecule from the protein by size-exclusion chromatography or dialysis, the separation of the labeled protein from the unlabeled one is far more challenging and in most cases is not possible. To demonstrate this, samples with 20 μm His6-GFP and various amounts of [Co^II^NTA] were treated with 10 mm H_2_O_2_ for 1 hour and then applied to a Ni-NTA column where the protein was eluted with a linear imidazole gradient. We observed three well-separated protein peaks with different affinities to the column (Figure 3); peaks (a) and (b) with multiple and one NTA attached to the His6-tag by coordination to Co^3+^, respectively, have a lower affinity to the column, and peak (c) corresponds to unmodified His6-GFP. The distribution of the peaks changed depending on the initially used [Co^II^NTA] concentration; the higher the concentration, the larger peak (a) with multiple NTA was (Figure S8 in the Supporting Information). In control experiments we showed that the addition of 10 mm H_2_O_2_ or 800 μm of [Co^II^ NTA] alone to His6-GFP has no effect on the elution profile (Figure S9 in the Supporting Information). Similarly, the NTA-conjugated fluorophore coumarin-NTA was used to label His6-GFP by means of the Co^3+^ interaction with similar results (Figure S10 in the Supporting Information). Analysis of peak (b) in this labeling reaction by UV/Vis spectroscopy showed that in this peak the 1:1 complex is formed between coumarin-NTA and His6-GFP and that this complex is observed as a single peak in size-exclusion chromatography (Figure S11 in the Supporting Information). Thus, by taking advantage of the slow ligand-exchange rates of the Co^3+^ center, labeled and unlabeled His6-tagged proteins can be separated. The use of metals ions in the presence of H_2_O_2_ can cause Fenton reactions, which result in the formation of radicals and can cleave the protein backbone.^32^, ^33^ However, under the reaction conditions used we did not observe backbone cleavage by MALDI-TOF MS (Figure S12 in the Supporting Information).

**Figure 3:**
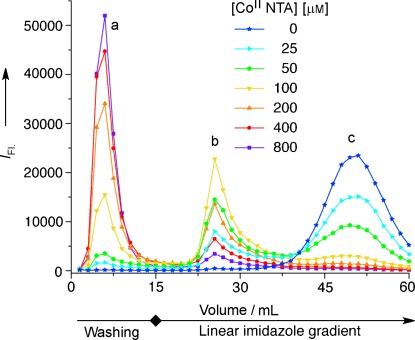
His6-GFP (20 μm), treated with various amounts of [Co^II^NTA] and 10 mm H_2_O_2_ for 1 hour, was passed through a Ni-NTA column and eluted with a linear imidazole gradient, and then the GFP fluorescence in each fraction was measured. His6-GFP conjugates with one (b) or multiple Co^III^ NTA units (a) could be separated from the His6-GFP (c).

In summary, we have demonstrated that Co^3+^ can mediate the interaction between the His6-tag and NTA and forms a complex that is inert towards ligand exchange, is thermodynamically stable, and does not react with chelators or reducing agents. Furthermore, the [Co^III^NTA(His6-protein)] complexes have a lower affinity towards Ni-NTA columns, which makes it possible to separate the complexes from the unmodified protein. The presented method is very practical due to the popularity of the His6-tag purification and the availability of many NTA-modified materials and molecules. Therefore, there are many possible applications of this system such as the stable immobilization of proteins on nanomaterials and surfaces as well as the labeling of proteins with fluorescent and other spectroscopically active molecules. The requirement of an oxidizing agent to form the Co^3+^ center limits its applications to proteins that are not sensitive to oxidation such as extracellular proteins. In future studies, the ligands around the cobalt center could be optimized so that the oxidation is facilitated.

## Notes

As a service to our authors and readers, this journal provides supporting information supplied by the authors. Such materials are peer reviewed and may be re-organized for online delivery, but are not copy-edited or typeset. Technical support issues arising from supporting information (other than missing files) should be addressed to the authors.
