# Supplementary material for: Cobalt(III) as a Stable and Inert Mediator Ion between NTA and His6-Tagged Proteins
Source: Angew Chem Int Ed Engl. 2013 Jun 5;52(29):7593–6. doi: 10.1002/anie.201210317 (PMC3798096; doi:10.1002/anie.201210317)
Supplement: Supplementary file 1 [file anie0052-7593-sd1.pdf]

Angewandte Chemie
Eine Zeitschrift der Gesellschaft Deutscher Chemiker

Supporting Information

© Wiley-VCH 2013

69451 Weinheim, Germany

Cobalt(III) as a Stable and Inert Mediator Ion between NTA and His6-Tagged Proteins**

*Seraphine V. Wegner and Joachim P. Spatz**

anie_201210317_sm_miscellaneous_information.pdf

Experimental Details

1. Expression and purification of His6-GFP

The His6-GFP expression plasmid (Addgene # 29663)[1] was transformed into BL21(DE3) *E.coli* cells. A 10 ml overnight pre-culture started from single colony was added to 1 L of freshly autoclaved LB medium with 50 mg kanamycin. The cells were grown to OD_{600} = 0.6 at 37 °C at 250 rpm and then protein expression was induced with 0.5 mM IPTG and the cells were grown for another 4 hours. Cells were harvested by centrifugation at 5000 rpm for 10 min. The cell pellet was suspended into 20 ml buffer A (50 mM Tris-HCl [pH 7.4], 300 mM NaCl). The cells were lysed by sonication and the lysate was cleared by centrifugation at 12000 rpm for 30 min followed by filtration through a 0.45 μm filter. The lysate was incubated with 2 ml of Ni-NTA Agarose beads for 1 hour. Then the beads were washed with 50 ml buffer C (Buffer A with 25 mM imidazole) and the protein was eluted with 10 ml buffer B (Buffer A with 250 mM imidazole). The purified GFP-His6 was dialyzed against 2 L buffer A twice for at least 6 hours. The His6-MPB (N-terminal His6-tag maltose binding protein) (Addgene # 29656) was expressed using the same protocol as for the His6-GFP. His6-TEV (N-terminal His6-tag tobacco etch virus protease), His6-transportin (N-terminal His6-tag human transportin 1) and His6-SUMO1 (N-terminal His6-tag small ubiquitin-like modifier 1) were generously donated Dr. Sakin.

2. Study of Co^{3+} mediated GFP-His6 binding to NTA agarose beads

Preparation of Co^{2+}-NTA agarose beads with immobilized His6-GFP. 1.75 ml of Ni-NTA agarose resin (Novagen, 2.39×10^9 beads/ml, 5.3×10^7 NTA groups/bead) was treated with the solutions listed below in the order given, and between each step the bead suspension was centrifuged for 1 min at 2000 rpm to sediment the beads and the liquid was decanted. 1) 15 ml Mili-Q water, 2) 5 ml 0.1 M EDTA (pH 7.5), 3) 3 × buffer A, 4) 2 ml 0.1 M $CoCl_2$, 5) 15 ml buffer B, 6) 3 × buffer A. Next, the Co^{2+} loaded NTA agarose beads were incubated with an equal volume of 10 μM His6-GFP solution in buffer A.

Optimization of H_2O_2 treatment for the oxidation to Co^{3+}. 100 μl aliquots of the above described bead suspension were added to 1.5 ml tubes for each experiment. Then 50 μl of a H_2O_2 solution in buffer A was added to each tube to result in 0, 0.1, 0.2, 0.5, 1, 2, 5, 10, 20, 50, 100 mM H_2O_2 final concentrations and the beads were reacted with the H_2O_2 for 1 hour. Then 50 μl of a 1 M imidazole stock solution (final imidazole concentration= 250 mM) was added to each tube and 100 μl of the each solution above the beads was analyzed for GFP fluorescence (λ_{ex}= 480 nm, λ_{em}= 510 nm) (TECAN, infinite 2000 plate reader). The amount of His6-GFP which eluted from the sample that was not treated with H_2O_2 was

defined as 100 % and all the His-GFP in solution and on the beads was calculated accordingly (Figure 2, Supporting Information, Figure S1). The solutions were added back to the beads after the fluorescence measurements and the fluorescence was measured again after 1, 3, 7, and 16 days to analyze the release of GFP-His6 from Co^{3+}-NTA beads over time. The initial rate of Hi6-GFP release from the beads was fitted to 1st order kinetics using the data from the samples that were treated with 5, 10 and 20 mM H_2O_2 (Supporting Information, Figure S4). All experiments were done in duplicate.

To show the generality of the presented concept, aliquots of Co^{2+}-NTA agarose beads, prepared as described above, were incubated with an equal volume of various His6-tagged protein solutions (10 μM). 100 μl aliquots of these beads were incubated with 20 mM H_2O_2 for 1 hour before imidazole was added to a final concentration of 250 mM. The solution above the beads was analyzed for eluted protein by SDS-PAGE gel (Invitrogen, NuPage Bis-Tris 4-12 %) (Supporting Information, Figure S3).

Chemical stability measurements of the Co^{3+}-NTA. To obtain Co^{3+}-NTA agarose beads with immobilized His6-GFP, the above-described immobilized His6-GFP on Co^{2+}-NTA agarose beads were reacted with 20 mM H_2O_2 for 1 hour. Then the excess H_2O_2 was removed by repeated washing of the beads with buffer A. Twice the bead volume of buffer A was then added to the beads and 150 μl aliquots of this suspension were used in each experiment. For comparison an equal amount of Co^{2+}-NTA with GFP-His6 in an equal volume of buffer A was used as well. 50 μl of the appropriate chelators (final concentrations: 250 mM imidazole, 25 mM NTA and 25 mM EDTA) or reducing agents (final concentration: 1 mM) in combination with 250 mM imidazole was then added to the beads and left to react for 1 hour (Figure 3). 100 μl of the solution above the beads was then analyzed for the His6-GFP fluorescence with the same settings as above. The solutions were added back to the beads after the measurements and analyzed 24 hours later again (Supporting Information, Figure S5). All experiments were done in duplicate. Further, equal amounts of Co^{2+}-NTA and Co^{3+}-NTA beads were treated with 100 mM ascorbate, TCEP and DTT in the presence of 250 mM imidazole or put into pH 3.5 acetate buffer. The amount of eluted protein after 1 hour and 1 day was quantified by His6-GFP fluorescence where possible and SDS-PAGE gels (Invitrogen, NuPage Bis-Tris 4-12 %) (Supporting Information, Figure S6).

Stability of His6-GFP immobilized at Co^{3+}-NTA centers in cell culture. REF52 cells were kept under standard cell culture conditions in 10 % FBS-DMEM medium in 48 well cell culture plates. To a 70 % confluent REF52 culture, equal amounts of His6-GFP immobilized onto NTA agarose beads at Ni^{2+}, Co^{2+} and Co^{3+} centers in DMEM were added (250 μl DMEM with 50 μl bead volume). The medium with the beads was removed from the culture plate after 1, 6 and 24 hours, 250 mM imidazole was added and the His6-GFP fluorescence in the supernatant was measured. The fluorescence intensity measured for the Ni^{2+} beads was set to 100 % (Supporting Information, Figure S7).

3. UV-Vis and ESI analysis of Co^{2+} and Co^{3+} complexes with His6-peptide and NTA

Different complexes were obtained by mixing the ligands in the corresponding stoichiometry with 1.5 mM $CoCl_2$ in buffer A to obtain [Co^{II}(NTA)], [Co^{II}(NTA)(Imidazole)$_2$] and [Co^{II}(NTA)(His6-peptide)]. His6-peptide was purchased from Innovagen, with an acetyl functionalization on the N-terminus. To obtain the Co^{3+} versions of these complexes 20 mM H_2O_2 was added to these solutions and reacted for 1 hour. The UV-Vis spectra of these complexes were recorded and the maxima were determined (Supporting Information, Figure S2). For ESI analysis [Co^{II}(NTA)(His6-peptide)] was obtained by mixing the ligands in the corresponding stoichiometry with 2 mM $CoCl_2$ in 10 mM Tris-HCl [pH 7.4] and [Co^{III}(NTA)(His6-peptide)] was obtained by treatment with 20 mM H_2O_2 for 1 hour. Negative-ion mode ESI: M^- = [Co^{II}(NTA)(His6-peptide)]$^-$ theoretical (M^-): 1129.33 g/mol, observed m/z: 1129.33 (M^-). M= [Co^{III}(NTA)(His6-peptide)] theoretical: 1129.33 g/mol (M), observed m/z: 1145.75 (M + OH^-), 1238.93 (M + TFA^-)

4. Separation of different His6-GFPspecies labeled via NTA by Ni-NTA columns

Co^{3+} mediated labeling of His6-GFP with NTA. Aliquots of 250 µl with 20 µM His6-GFP and various amounts of [Co^{II} NTA] (0-800 µM) were incubated with 10 mM H_2O_2 for 1 hour before they were applied to a 5 ml Ni-NTA column connected to a FPLC system (AKTA Purifier). The column was run with 2.5 ml/min flow and 1.5 ml fractions were collected. The column was first washed with 10 ml buffer A and then a linear imidazole gradient up to 100 mM imidazole over 50 ml was used to elute different His6-GFP species. Then, 200 µl from each fraction was analyzed for Hi6-GFP fluorescence (Figure 4). As controls His6-GFP alone and GFP-His6 in the presence of 800 µM [Co^{II} NTA] were also analyzed in the same way as the H_2O_2 treated samples (Supporting Information, Figure S9). The distribution of the peaks was analyzed by integrating the area of each peak and setting the total area under the peaks to 100 % for each run (Supporting Information, Figure S8). To ensure that His6-GFP is not damaged by Fenton reactions in the presence of [Co^{II} NTA] (800 µM) before and after incubation with 10 mM H_2O_2 for 1 hour, these samples are analyzed by MALDI-TOF (Supporting Information, Figure S12). As a positive control His6-GFP (20 µM) was incubated with 200 µM Co^{2+}, 4.6 mM ascorbate and 10 mM H_2O_2, a conditions where Fenton reactions are known to take place.[2]

Synthesis of coumarin-NTA. 7-hydroxycoumarin-3-carboxylic acid *N*-succinimidyl ester (5 mg, 16.5 nmol) was dissolved in 100 µl DMF and added dropwise to a solution of N_α,N_α-bis(carboxymethyl)-L-lysine hydrate (8.6 mg, 33 nmol) in 0.3 M HEPES pH 7.4. The reaction was stirred for 30 min. at RT and the purified by C18 reverse phase column (30 % Acetonitrile 0.1 % TFA, flow rate). The purified compound was lyophilized and the product was characterized by MALDI-TOF and ^{1}H-NMR. Molecular

formula: $C_{20}H_{22}N_2O_{10}$ Calculated: 450.13 g/mol Found: 451.26 g/mol. ^1H NMR (300 MHz, DMSO-d_6): 8.75 (s, 1 H), 8.60 (t, 1H, J = 6.0 Hz), 7.79 (d, 1 H, J= 8.7 Hz), 6.86 (dd, 1H, J=8.7 Hz J=2.1 Hz), 6.78 (d, 1 H, J= 1.8 Hz), 3.4-3.2 (m, 7 H), 1.5-1.3 (m, 6H).

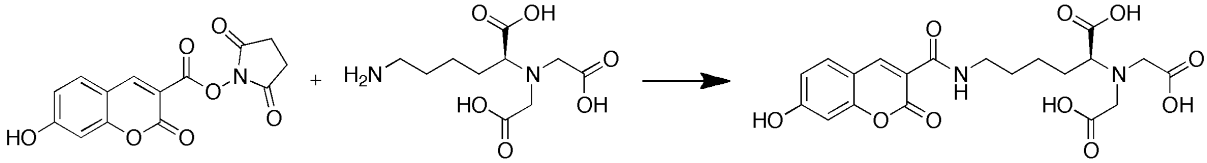

Scheme S1. Synthesis of coumarin-NTA.

Co^{3+} mediated labeling of His6-GFP with coumarin-NTA. Aliquots of 250 µl with 20 µM His6-GFP with various amounts of [Co^{II}NTA-coumarin] (0-400 µM) were incubated with 10 mM H_2O_2 for 1 hour before they were applied to a HiTrap Desalting column (GE Bio-sciences, CV 5 × 5ml, Buffer A with 2.5 ml/min, fraction size 1 ml over 30 ml). The fraction with the highest His6-GFP fluorescence (Fraction 9) was then applied to the Ni-NTA column and run with the same gradient as described above. The fluorescence of each fraction was recorded both for the His6-GFP and coumarin-NTA (λ_{ex}= 386 nm, λ_{em}= 448 nm) signal and plotted (Supporting Information, Figure S10). In a large scale reaction, 500 µl of 80 µM His6-GFP and 200 µM [Co^{II}NTA-coumarin] were incubated with 10 mM H_2O_2 for 1 hour, then the mixture was purified by Ni-NTA column and peak (b) was concentrated with a 10 kDa centrifugal concentrating device. The UV-VIS spectrum of the concentrated peak (b) was recorded and the His6-GFP (ε_{400}= 16000 $M^{-1}cm^{-1}$, ε_{485}= 83300 $M^{-1}cm^{-1}$) and coumarin-NTA (ε_{400}= 36000 $M^{-1}cm^{-1}$) concentrations in the sample were calculated (Supporting Information, Figure S11a). The concentrated sample was also applied to a HiTrap Desalting column (GE Bio-sciences, CV 5 × 5ml, Buffer A with 2.5 ml/min, fraction size 1 ml over 30 ml) using detection at 280 nm, 400 nm and 490 nm. Further, the fluorescence of each fraction was measured both for His6-GFP and coumarin-NTA (Supporting Information, Figure S11b).

References:

[1] J.-D. Pédelacq, S. Cabantous, T. Tran, T. C. Terwilliger, G. S. Waldo, *Nat. Biotech.* **2005**, *24*, 79–88.

[2] M. Andberg, J. Jäntti, S. Heilimo, P. Pihkala, A. Paananen, A. M.P. Koskinen, H. Sönderlund, M. B. Linder, *Protein Sci.* **2007**, *16*, 1751-1761.

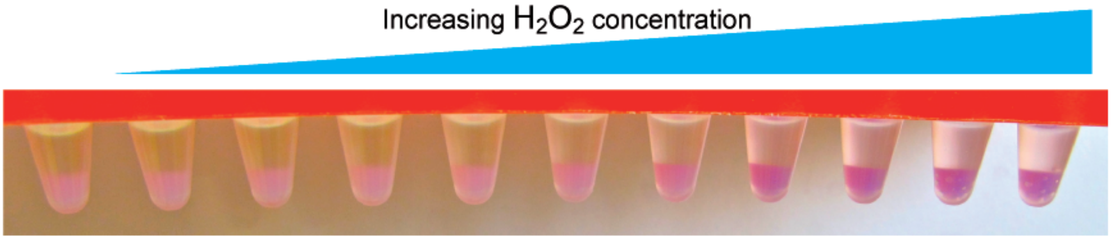

Figure S1. NTA agarose beads loaded with Co^{2+} and immobilized His6-GFP were incubated various concentrations of H_2O_2. The oxidation of Co^{2+} to Co^{3+} can be observed by the color change of the beads from pink to purple.

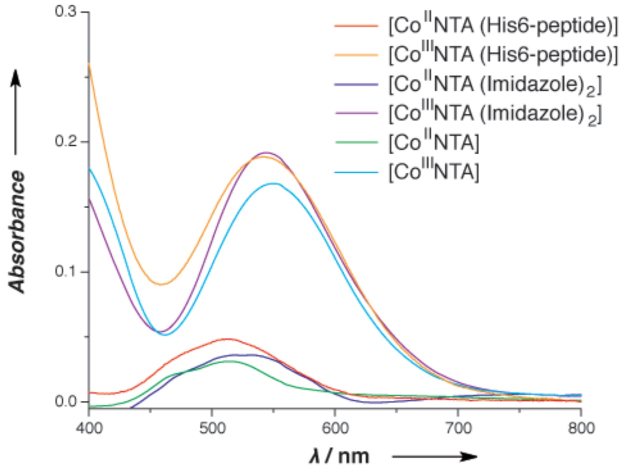

Figure S2. UV-VIS spectra of different Co^{2+} and Co^{3+} complexes with NTA in the absence and presence of His6-peptide and imidazole. The absorption maximum is slightly blue shifted when additional nitrogen donor ligands are added to [Co^{III} NTA] (λ_{max}=554 nm) to form [Co^{III} NTA(His6-peptide)] (λ_{max}=542 nm) and [Co^{III} NTA(Imidazole)$_2$] (λ_{max}=543 nm).

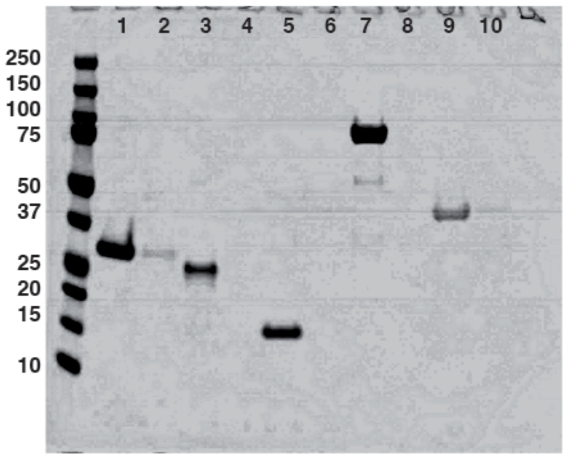

Figure S3. NTA agarose beads with different immobilized His6-tagged proteins at Co^{2+} (lane 1, 3, 5, 7, 9) and Co^{3+} (2, 4, 6, 8,10) centers were incubated with 250 mM imidazole and the protein in the solution above the beads was analyzed by SDS-PAGE gel. Lane 1-2: His6-GFP (29 kDa), lane 3-4: His6-TEV (26 kDa), lane 5-6: His6-SUMO1 (11 kDa), lane 7-8: His6-transportin (100 kDa), lane 9-10: His6-MBP (43 kDa). While the His6-tagged proteins bound to Co^{2+} centers elute from the beads, the proteins at Co^{3+} centers stayed immobilized.

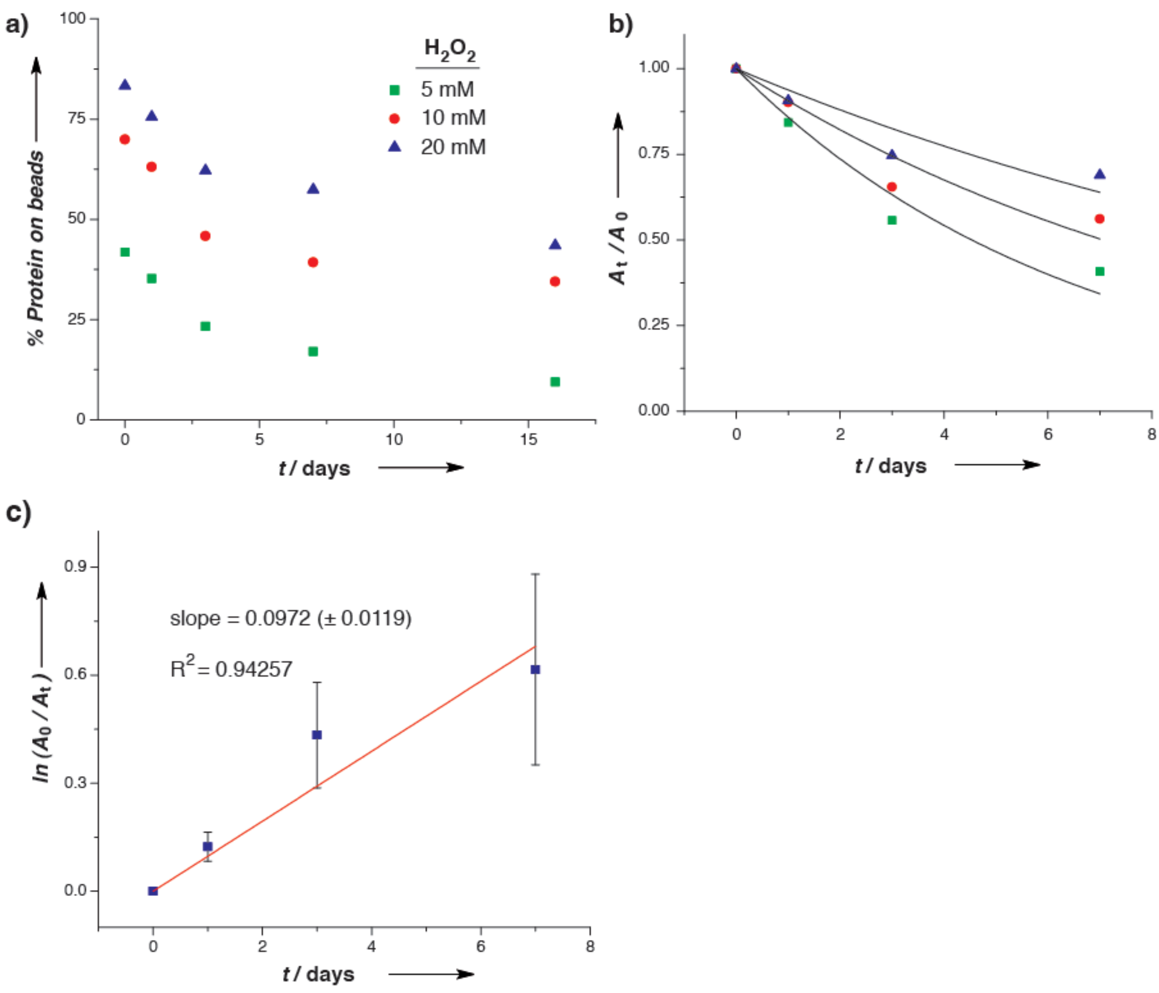

Figure S4. Kinetic data analysis of immobilized GFP-His6 dissociation from Co^{3+}-NTA agarose beads. a) Measurement of His6-GFP on the beads over 16 days in the presence of 250 mM imidazole. b) The initial rate of dissociation was fitted to first order kinetics for the 3 samples individually. The k_{off} for 5 mM, 10 mM and 20 mM H_2O_2 were found to be 0.15 (± 0.018) day^{-1} (R^2=0.9554), 0.098 (± 0.014) day^{-1} (R^2=0.9088) and 0.063 (± 0.011) day^{-1} (R^2=0.8519) respectively. c) The initial rate of dissociation in a) was fitted to first order kinetics for the 3 data sets. k_{off}= 0.0972 (± 0.0119) day^{-1} = 1.12 (± 0.14) ×10^{-6} s^{-1}. Systematic errors can rise from the reverse reaction where the His6-GFP binds back on the beads, the stability of the protein over this long incubation time and the approximation that the initial rate is equal to the rate over the first 7 days, which were used in the fitting.

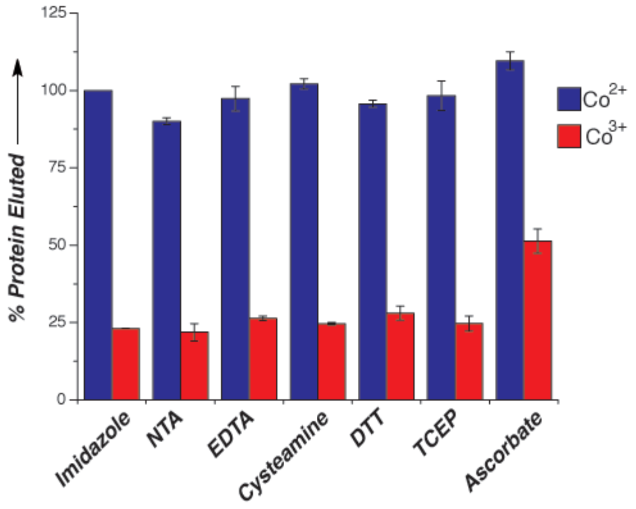

Figure S5. Chemical reactivity of the immobilized His6-GFP on Co^{3+}-NTA agarose beads. Co^{2+} and Co^{3+} NTA-agarose beads with immobilized His6-GFP are treated with the chelators imidazole (250 mM), NTA and EDTA (25 mM) or various reducing agents (1 mM) commonly used in protein chemistry in combination with imidazole (250 mM) for 24 hours. Due to the kinetic inertness of the Co^{3+}, only very little His6-GFP is eluted. Ascorbate is the only reducing agent that succeeds in reducing some of the Co^{3+} centers on the beads so that more His6-GFP is eluted.

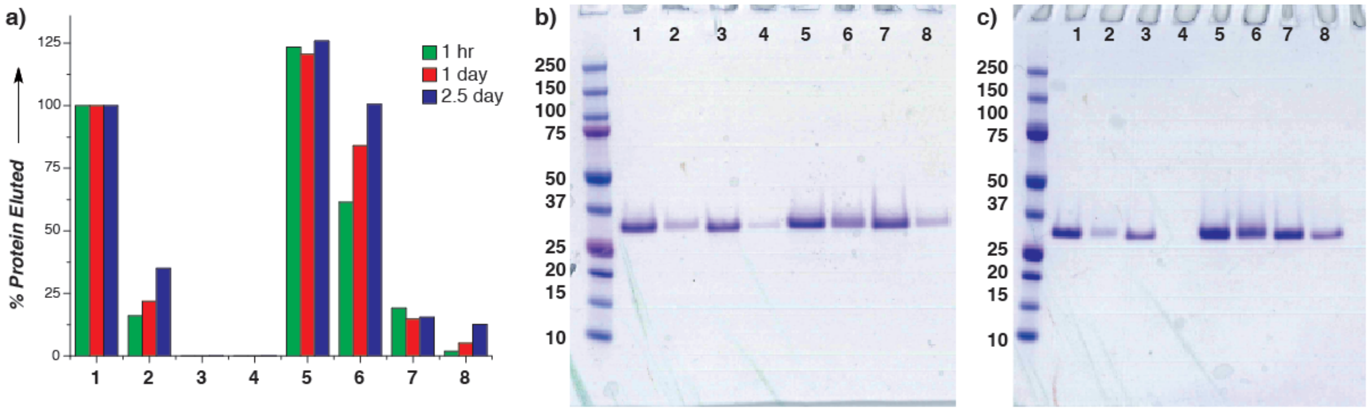

Figure S6. Chemical reactivity of immobilized His6-GFP on Co^{3+}-NTA agarose beads. The amount of His6-GFP in the supernatant, which eluted from the NTA agarose beads, was quantified by a) measuring the GFP fluorescence after 1 hour, 1 day and 3 days, and SDS-PAGE gel after b) 1 hour and c) 1 day. NTA agarose beads with immobilized His6-GFP at Co^{2+} (1, 3, 5, 7) and Co^{3+} (2, 4, 6, 8) centers were incubated 1-2) with 250 mM imidazole, 3-4) in 100 mM pH 3.5 acetate buffer, 5-6) with 100 mM ascorbate in the presence of 250 mM imidazole and 7-8) with 100 mM TCEP in the presence of 250 mM imidazole. As can be observed both from the GFP fluorescence and in the SDS-PAGE gel 100 mM ascorbate can almost entirely reduce the Co^{3+} centers, therefore the His6-GFP can be eluted with imidazole. While 100 mM TCEP partially can reduce the Co^{3+} centers, acidification of the Co^{3+} beads does not lead to protein elution.

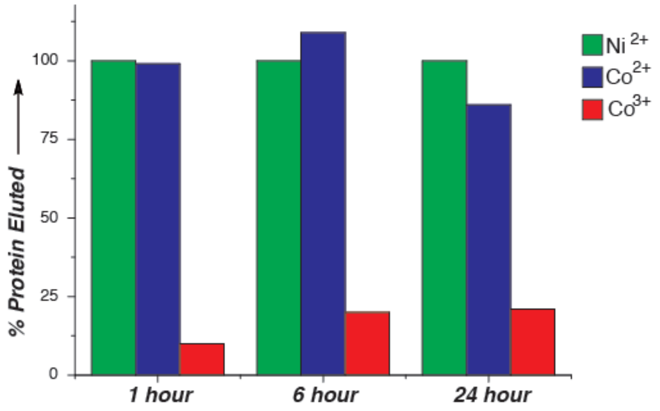

Figure S7. Stability of immobilized His6-GFP on Co^{3+}-NTA agarose beads in cell culture. NTA-agarose beads with His6-GFP immobilized at Ni^{2+}, Co^{2+} and Co^{3+} centers were placed into cultures of adherent REF52 cell cultures for 1, 6 and 24 hours before the beads were taken out with the medium and 250 mM imidazole was added. The amount of His6-GFP in the supernatant, which eluted from the NTA agarose beads, was quantified by measuring the GFP fluorescence. Even after 24 hours in cell culture, the His6-GFP immobilized at the Co^{3+} centers is still bound to the beads.

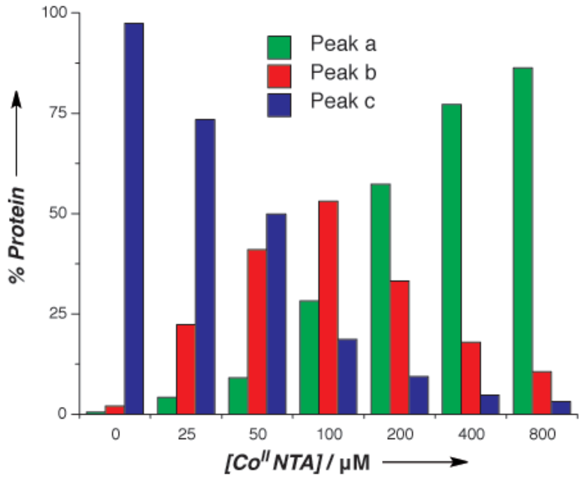

Figure S8. The distribution of His6-GFP species, which are labeled with different numbers of NTA groups, in the presence of various amounts of [Co^{II} NTA]. The total area under the chromatogram is set to 100 % and the % of each peak is calculated as the area under the respective peak.

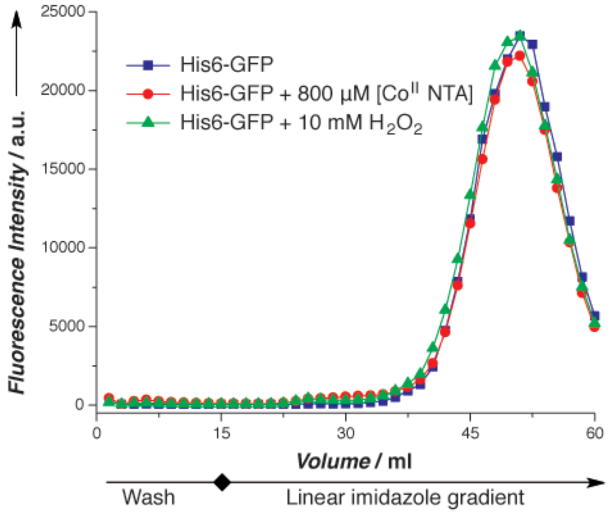

Figure S9. Control experiments for His6-GFP binding to the Ni-NTA column. The addition of 10 mM H_2O_2 or up to 800 µM [Co^{II} NTA] does not affect the binding of GFP-His6 to the Ni-NTA column.

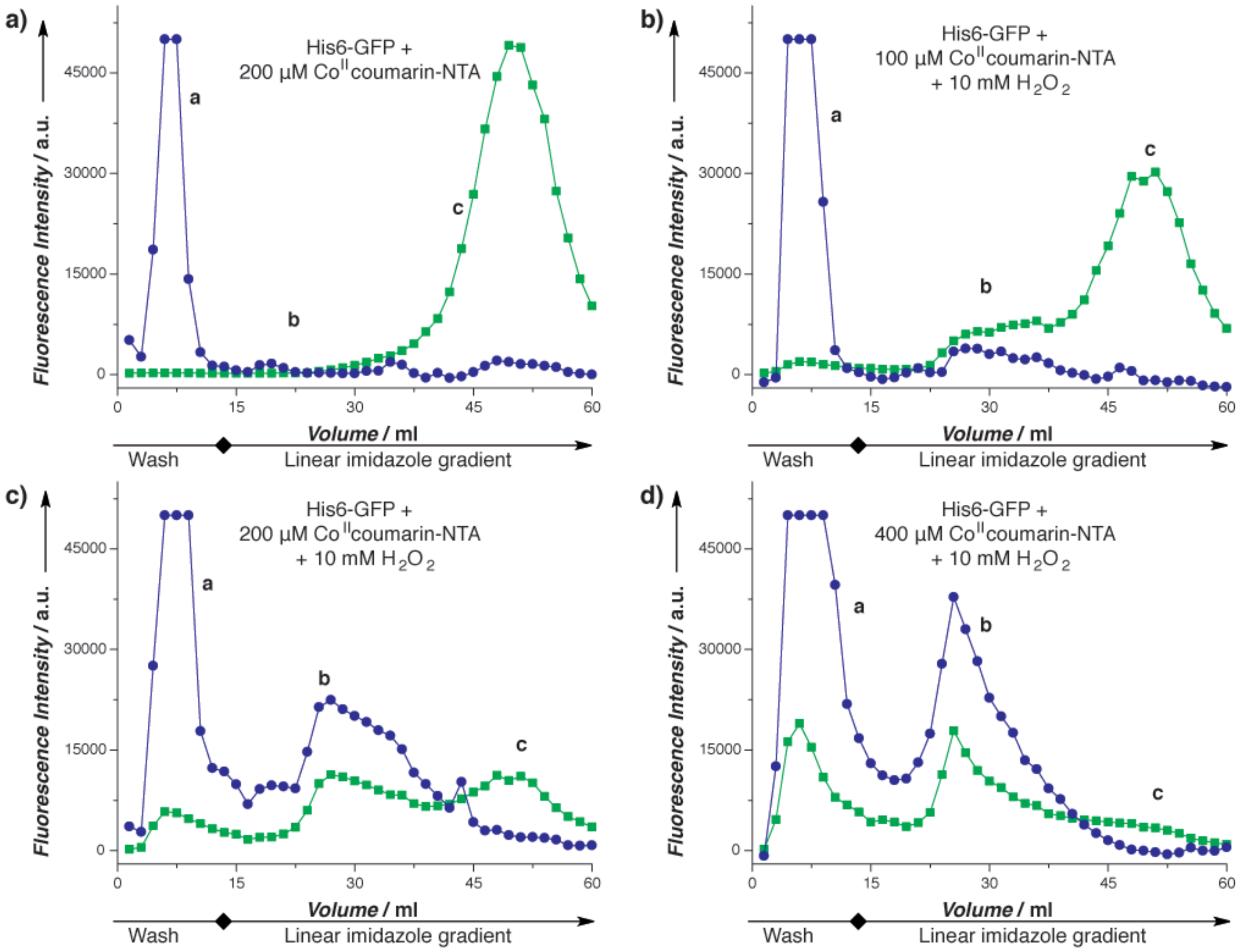

Figure S10. Aliquots of His6-GFP (20 µM) were incubated with various amounts of coumarin-NTA and Co^{2+} (a) 200 µM, b) 100 µM, c) 200 µM, and d) 400 µM) and then b-d were treated with H_2O_2 (10 mM) for 1 hour before all samples were run over a desalting column. Then the most concentrated protein fraction from the desalting column was applied to a Ni-NTA column, the protein was eluted with a linear imidazole gradient as described above and both the GFP and coumarin fluorescence was measured for each fraction. The **GFP** fluorescence is shown in **green** and the **coumarin** fluorescence in **blue**. Parallel to what is observed in Figure 4, the peak with the lowest affinity towards the Ni-NTA column gets bigger the higher the [Co^{II} NTA-coumarin] concentration initially used is. High coumarin fluorescence is even observed without H_2O_2 treatment in the early fractions in a) where there is no GFP signal. This indicates that there is some coumarin-NTA that binds nonspecifically to the protein and caries over even after the desalting column.

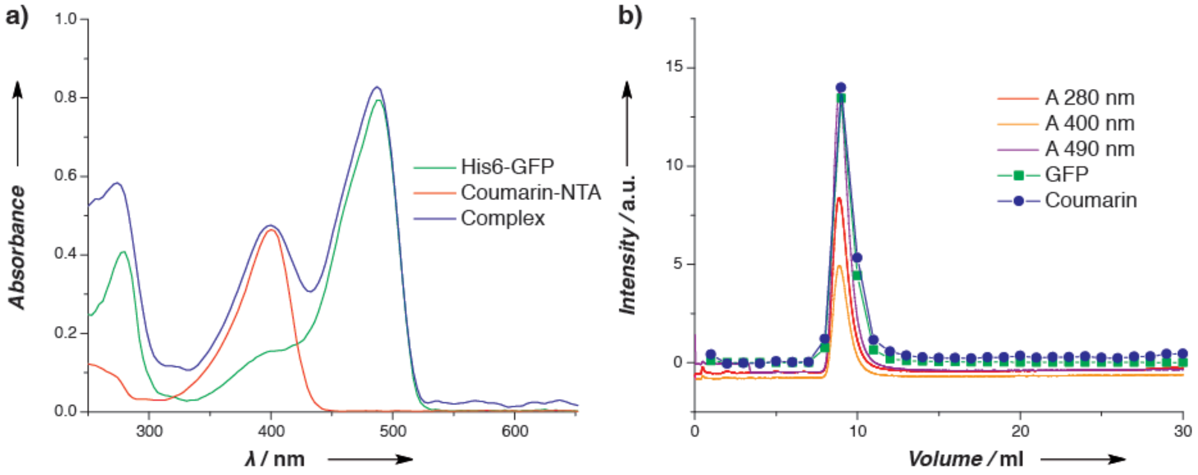

Figure S11. Peak (b) after the Ni-NTA purification from a labeling reaction of His6-GFP (80 µM) and [Co^{II} coumarin-NTA] (200 µM) incubated with H_2O_2 (10 mM) was concentrated. a) The UV-VIS spectra of the complex (peak b), His6-GFP (94 µM) and coumarin-NTA (130 µM) were recorded. In the complex His6-GFP and coumarin-NTA concentrations are calculated to be 96 µM and 91 µM respectively. Thus, the formed complex is approximately 1:1. b) The concentrated sample was run on a size exclusion column (CV= 25 ml, fraction size 1 ml) using detection at 280 nm, 400 nm and 490 nm. Afterwards all the fractions were also analyzed for their GFP and coumarin fluorescence. In the chromatogram signal from the coumarin and GFP were only detected at the same time. Thus, the Co^{3+} mediated binding of coumarin-NTA to His6-GFP is stable.

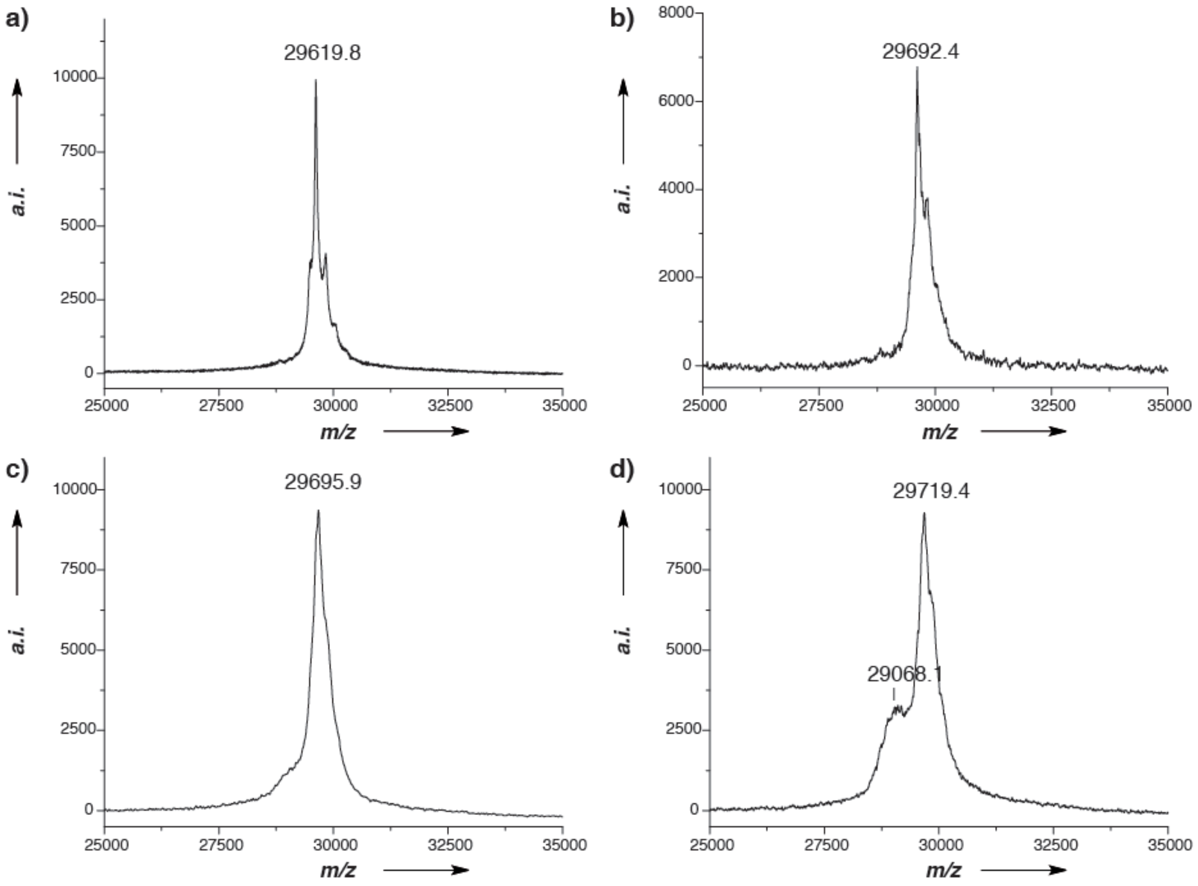

Figure S12. MALDI-TOF analysis of His6-GFP under the reaction conditions used for protein labeling. a) His6-GFP (20 µM), b) with 800 µM [Co^{II} NTA] before and c) after 1 hour H_2O_2 (10 mM) treatment. d) His6-GFP under reaction conditions where Fenton reactions takes place. (200 µM Co^{2+}, 4.6 mM ascorbate and 10 mM H_2O_2).[2] The theoretical MW of GFP with His6-tag and without His-tag is 29621 and 28567 g/mol respectively. In d) a shoulder peak is observed due to the cleavage of the His6-tag by Fenton reactions but this is not observed in the other sample despite the presence of [Co^{II} NTA] and H_2O_2.
